# Supplementary material for: Integrative Stacking Machine Learning Model for Small Cell Lung Cancer Prediction Using Metabolomics Profiling
Source: Cancers (Basel). 2024 Dec 18;16(24):4225. doi: 10.3390/cancers16244225 (PMC11727543; doi:10.3390/cancers16244225)
Supplement: Supplementary file 1 [file cancers-16-04225-s001.zip › cancers-3321183-supplementary-final.pdf]

Supplementary Materials

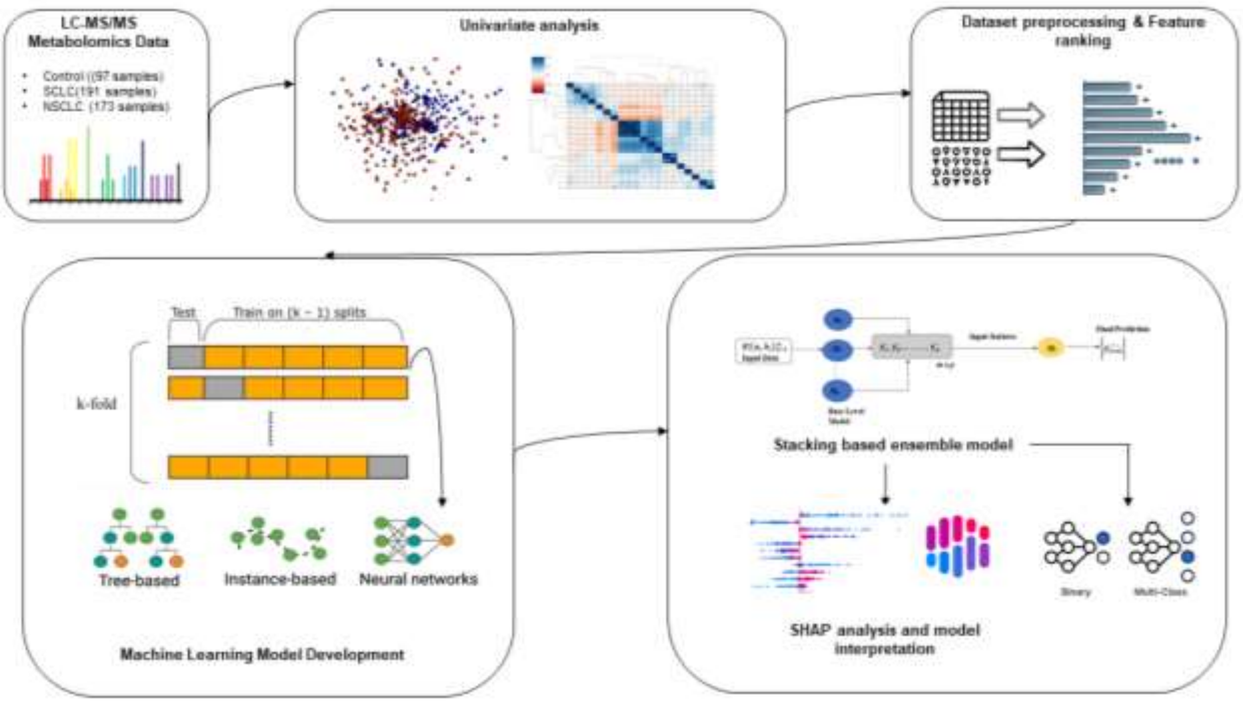

Supplementary Figure S1. Methodology Overview of the Study.

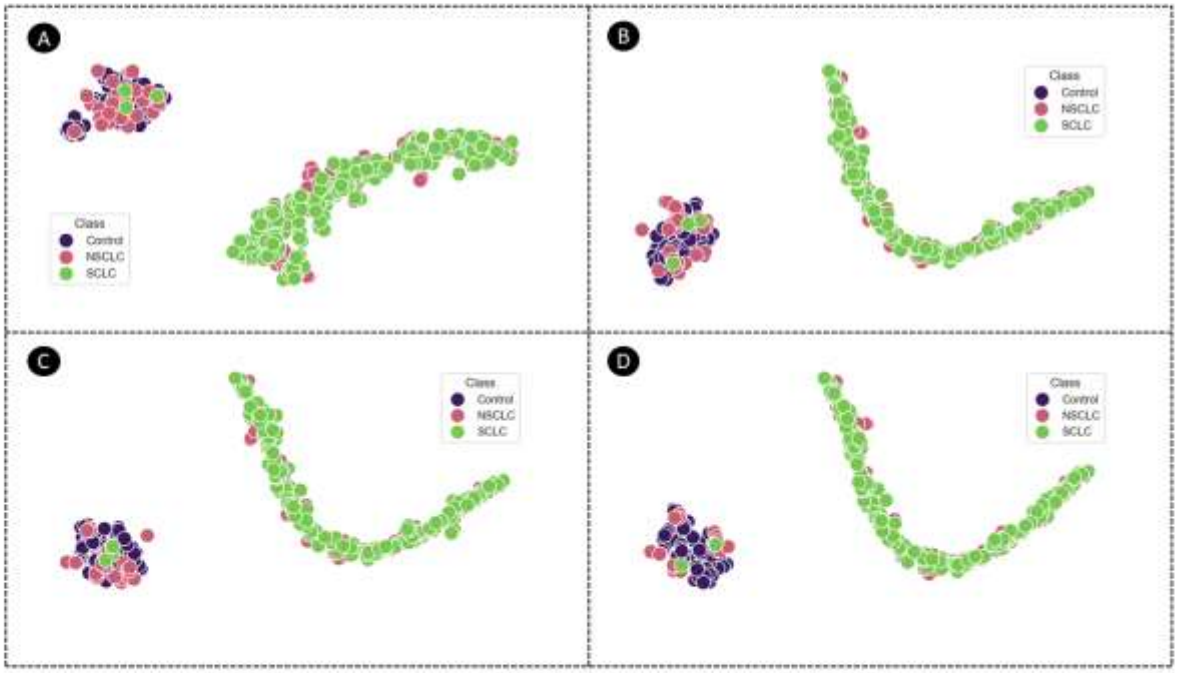

Supplementary Figure S2. t-SNE visualization for the negative ion data. (A) t-SNE visualization for all features. (B) t-SNE visualization for top 50 features. (C) t-SNE visualization for top 30 features. (D) t-SNE visualization for top 20 features.

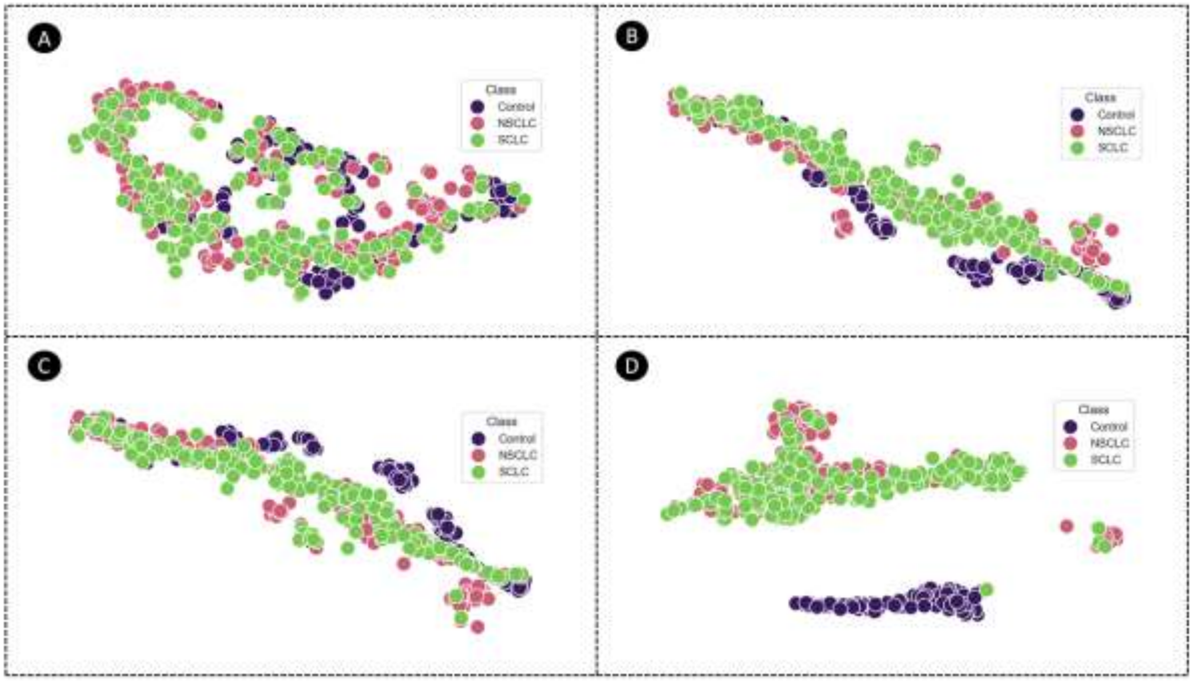

Supplementary Figure S3. t-SNE visualization for the positive ion data. (A) t-SNE visualization for all features. (B) t-SNE visualization for top 50 features. (C) t-SNE visualization for top 30 features. (D) t-SNE visualization for top 20 features.

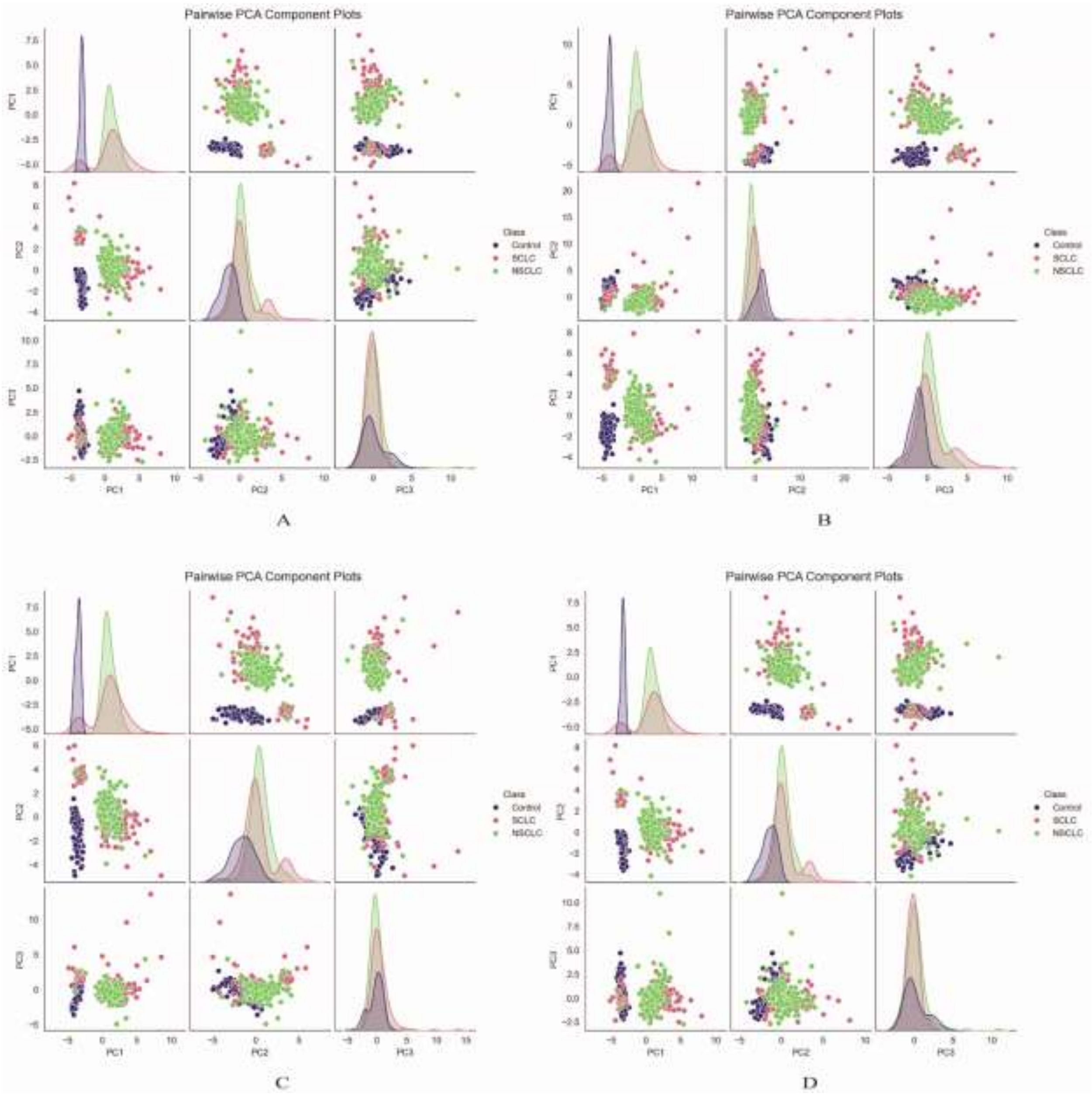

Supplementary Figure S4. PCA component plots for the negative ion data. This figure presents pairwise PCA component plots for all classes, illustrating the distribution and relationships between PC1, PC2, and PC3, The diagonal plots display the kernel density estimates (KDEs) of each principal component, highlighting how the data is distributed for individual classes. (A) For all features. (B)For top 50 features. (C) For top 30 features. (D) For the top 20 features.

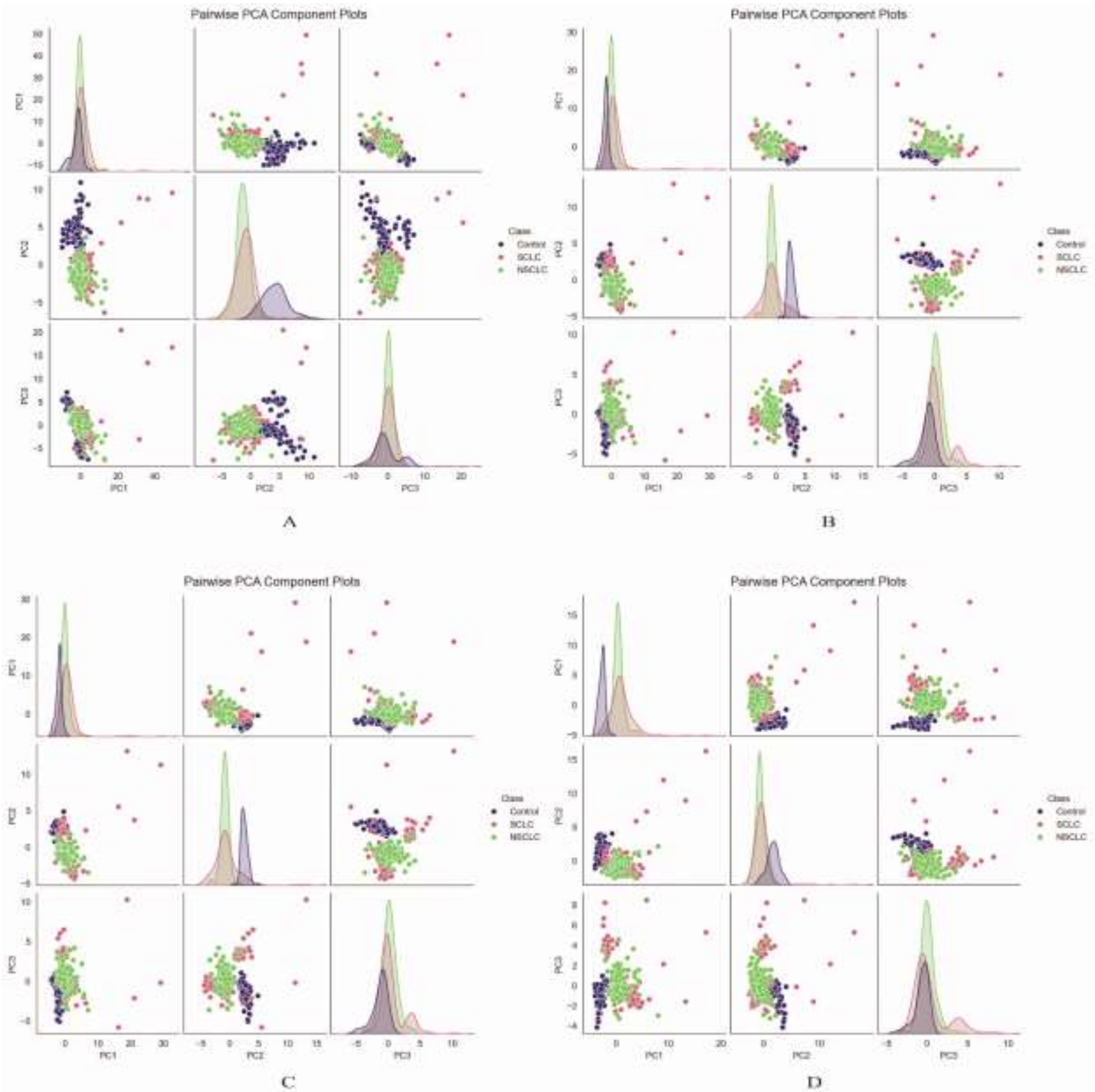

Supplementary Figure S5. PCA component plots for the Positive ion data. This figure presents pairwise PCA component plots for all classes, illustrating the distribution and relationships between PC1, PC2, and PC3, The diagonal plots display the kernel density estimates (KDEs) of each principal component, highlighting how the data is distributed for individual classes. (A) Includes all features. (B) For the top fifty features. (C) For the top thirty features. (D) For the top twenty features.

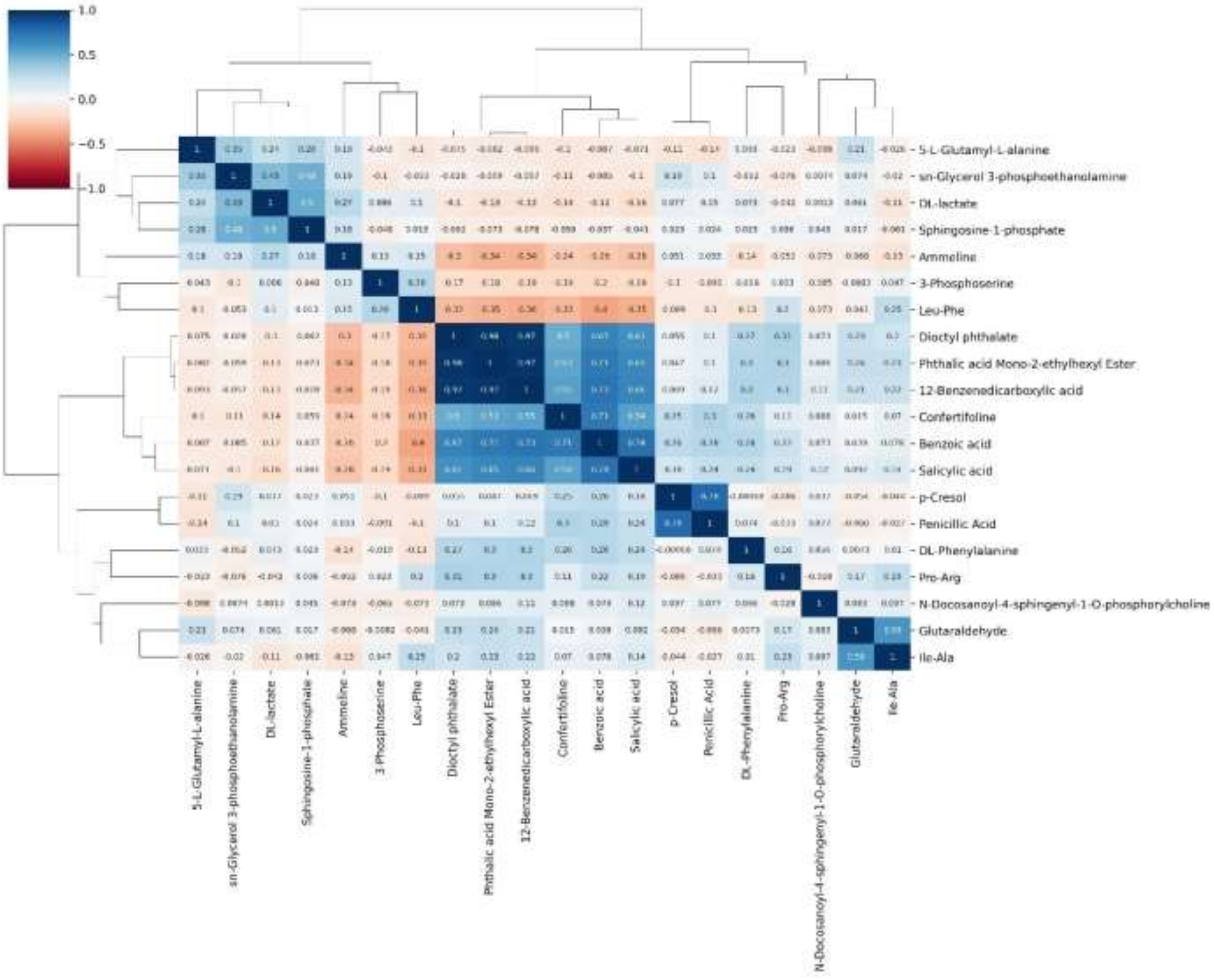

Supplementary Figure S6. Cluster Heatmap Depicting the Distinction Between SCLC and NSCLC Classes.

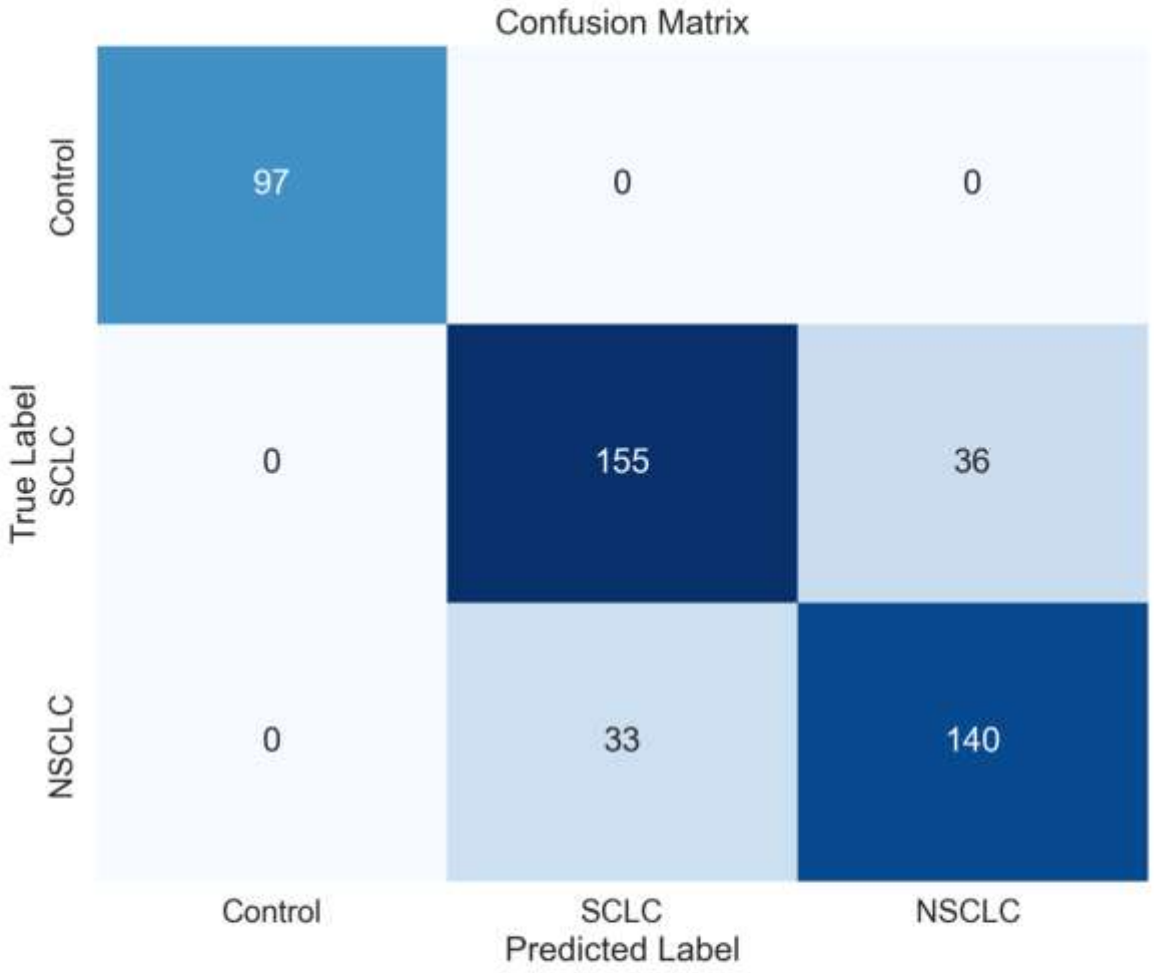

Supplementary Figure S7. Confusion matrix for the stacking-based SVM classifier in multi-class classification.

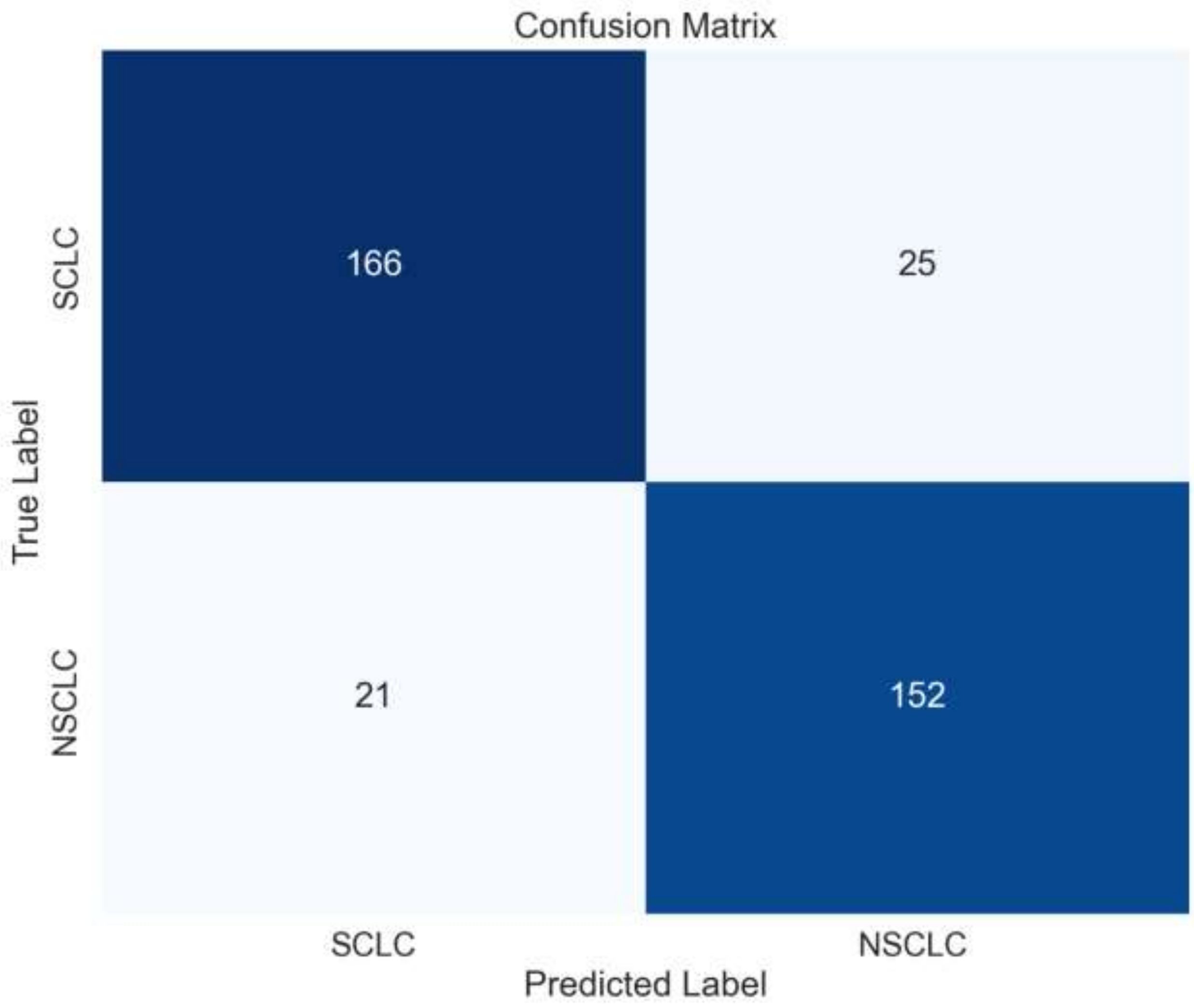

Supplementary Figure S8. Confusion matrix for the stacking-based ExtraTrees classifier in binary classification.

Supplementary Table S1. Comprehensive clinical characteristics.

**Total Sample (461)**			
**Variables**	**Controls (n = 97)**	**SCLC (n = 191)**	**NSCLC (n = 173)**
**Sex**			
Male	52	135	117
Female	45	56	56

**Age, years**			
<65	45	115	89
≥65	52	76	84
**Medication history**			
Yes	NA	52	63
No		139	110
**Drinking**			
Yes	NA	70	51
No		121	122
**Smoking**			
Yes	NA	111	80
No		80	93
**Histology**			
SCLC	NA	191	
squamous			82
adenocarcinoma			91
**NSE (ng/ml)**			
Median	NA	41.47	14.515
Range		3.4-740	2.9-95.87
**ProGRP (pg/ml)**			
Median	NA	1951	47.8
Range		10.7-39040	3-85
**Stage**			
Limited stage	NA	87	NA
Extensive stage		104	

NSE: neuron-specific enolase; SCLC: small cell lung cancer; NSCLC: non-small cell lung cancer;

Supplementary Table S2. Parameters of the MLPClassifier.

**Parameter**	**Value**
**activation**	**relu**
Final activation fiunction	*Softmax (multiclass)/sigmoid(binary) function*
hidden_layer_sizes	(13, 13)
solver	adam
learning_rate_init	0.001
max_iter	500
shuffle	False
random_state	111
tol	0.0001
batch_size	auto
momentum	0.9
early_stopping	False

Supplementary Table S3. Parameters of the MLPClassifier for Multi-Class Classification

**Parameter**	**Value**
activation	relu
Final activation fiunction	*Softmax (multiclass)/sigmoid(binary) function*
hidden_layer_sizes	(50, 50)
solver	adam
learning_rate_init	0.001
max_iter	500
shuffle	True
random_state	111
tol	0.0001
batch_size	auto
momentum	0.9
early_stopping	False

Supplementary Section S1: Evaluation Metrics

The seven-evaluation metrics—weighted recall (or sensitivity), specificity, precision, overall accuracy, and F1 score—are mathematically expressed in Equations 1 to 7.

$$\text{Accuracy}_{\text{class}_j} = \frac{TP_j+TN_j}{TP_j+TN_j+FP_j+FN_j} \quad (1)$$

$$\text{Precision}_{\text{class}_j} = \frac{TP_j}{TP_j+FP_j} \quad (2)$$

$$\text{Recall}_{\text{class}_j} = \frac{TP_j}{TP_j+FN_j} \quad (3)$$

$$\text{F1\_score}_{\text{class}_j} = 2 \times \frac{\text{Precision}_j \times \text{Recall}_j}{\text{Precision}_j + \text{Recall}_j} \quad (4)$$

$$\text{Specificity}_{\text{class}_j} = \frac{TN_j}{TN_j+FP_j} \quad (5)$$

$$\text{Positive predictive value }_{\text{class}_j} = \frac{TP_j}{TP_j + FP_j} \quad (6)$$

$$\text{Negative predictive value }_{\text{class}_j} = \frac{TN_j}{FN_j + TN_j} \quad (7)$$

Where, TP, FP, TN, and FN stand for the number of true positives, false positives, true negatives, and false negatives, respectively; and j is an index representing the classifier being evaluated.

Supplementary Section S2: Model Development

Algorithm 1 represents the overall process of our proposed model.

Algorithm 1: Development of a Stacking ensemble model.

Training Data $B = \{(a_j, b_j)\}_{j=1}^{n}$

$Output: A\ stacking\ Classifier\ (M_c)$

1. Step 1: Train the base-level classifier
2. For each $t = 1$ to T:
3. Train h_t using the training data B
4. end for
5. Step 2: Create a new dataset based on the predictions of the base-level classifiers:
6. For $j = 1$ to $n\ do$:
7. $B_h = \{a_{j'}, b_{j'}\}, where\ a_{j'} = \{ h_1(a_j), h_1(a_j), \dots, h_T(a_j) \}$
8. end for

9. Step 1: Train the stacking classifier using the meta-models.
10. Train stacking classifier M_c based on B_h
11. Return M_c
